# Disease-Specific as Well as Generic Quality of Life Is Widely Impacted in Autoimmune Hypothyroidism and Improves during the First Six Months of Levothyroxine Therapy

**DOI:** 10.1371/journal.pone.0156925

**Published:** 2016-06-03

**Authors:** Kristian Hillert Winther, Per Cramon, Torquil Watt, Jakob Bue Bjorner, Ola Ekholm, Ulla Feldt-Rasmussen, Mogens Groenvold, Åse Krogh Rasmussen, Laszlo Hegedüs, Steen Joop Bonnema

**Affiliations:** 1 Department of Endocrinology and Metabolism, Odense University Hospital, Odense, Denmark; 2 Department of Clinical Research, Faculty of Health Sciences, University of Southern Denmark, Odense, Denmark; 3 Department of Medical Endocrinology, Copenhagen University Hospital Rigshospitalet, Copenhagen, Denmark; 4 Department of Internal Medicine, Gentofte Hospital, Copenhagen, Denmark; 5 Department of Public Health, University of Copenhagen, Copenhagen, Denmark; 6 Optum Insight Inc, Eden Prairie, MN, United States of America; 7 National Institute of Public Health, University of Southern Denmark, Copenhagen, Denmark; 8 Department of Palliative Medicine, Bispebjerg University Hospital, Copenhagen, Denmark; Tel Aviv Sourasky Medical Center, ISRAEL

## Abstract

**Background:**

Hypothyroidism is often diagnosed, and subsequently treated, due to health-related quality of life (HRQL) issues. However, HRQL following treatment has never previously been assessed in longitudinal descriptive studies using validated instruments.

**Objective:**

To investigate disease-specific (ThyPRO) and generic (SF-36) HRQL, following levothyroxine therapy in patients with hypothyroidism due to autoimmune thyroiditis.

**Methods:**

This prospective cohort study was set at endocrine outpatient clinics at two Danish university hospitals. Seventy-eight consecutive patients were enrolled and completed HRQL questionnaires before, six weeks, and six months after initiation of levothyroxine therapy. Normative ThyPRO (n = 739) and SF-36 (n = 6,638) data were available for comparison and changes in HRQL following treatment were estimated and quantified.

**Results:**

Prior to treatment, all ThyPRO scales were significantly impacted (p<0.0001), compared to the general population sample. The same was observed for seven of eight SF-36 scales, the exception being Bodily Pain. Tiredness (ThyPRO) and Vitality (SF-36) were the most markedly impacted scales. After six weeks of treatment, nine of thirteen ThyPRO scales had significantly improved. ThyPRO improvements were consistent at six months, where five of eight SF-36 scales had also significantly improved, but deficits persisted for a subset of both ThyPRO and SF-36 scales.

**Conclusions:**

In this population of hypothyroid patients, HRQL was widely affected before treatment, with tiredness as the cardinal impairment according to both ThyPRO and SF-36. Many aspects of HRQL improved during the first six months of LT4 therapy, but full recovery was not obtained. Our results may help clinicians inform patients about expected clinical treatment effects.

## Introduction

Chronic autoimmune or Hashimoto’s thyroiditis (AIT) is common, and accounts for 85% of all cases of overt hypothyroidism in Denmark, where the overall annual incidence is approximately 47.2/100,000 [1,2]. The standard treatment is life-long levothyroxine (LT4) therapy, adjusting the dosage to achieve normal circulating thyrotropin (TSH) levels [3]. However, recent investigations suggest that LT4 cannot ensure a euthyroid state in all tissues simultaneously [3], and cross-sectional studies have reported impaired psychological well-being and cognitive functioning in euthyroid AIT patients on LT4 [4–7]. Health-related quality of life (HRQL), measured by reported outcomes (PROs), is increasingly used when evaluating treatment effects in clinical studies and practice [8]. Instruments can be divided into disease-specific and generic questionnaires. Combining the two types of measurement maximizes precision and sensitivity, and allows for comparability and interpretation across different patient groups and populations [9,10]. Measurement properties have been well-described for the most widely used generic instrument, SF-36 [11,12], while some currently available disease-specific instruments (4,6) have undergone initial validation in hypothyroid patients [6,13,14]. To further improve the measurement of HRQL in thyroid diseases, our group developed and validated the disease-specific ThyPRO questionnaire [15–20]. In AIT, HRQL has never previously been measured in descriptive longitudinal studies, and ThyPRO was chosen for this purpose since it is the only HRQL instrument that has been validated for responsiveness to treatment [21].

The objective of this prospective cohort study was therefore to investigate disease-specific (ThyPRO) and generic (SF-36) HRQL in patients with AIT before, during, and after six months of medical therapy with LT4, comparing their HRQL with that found in representative general population samples.

## Materials and Methods

### Design, setting and participants

This study was a prospective cohort study carried out from October 2008 to May 2012. Patients referred for hypothyroidism were consecutively recruited from the endocrine outpatient clinics at Copenhagen University Hospital, Rigshospitalet, and Odense University Hospital. The inclusion criteria were ≥18 years of age; indication for LT4 therapy treatment (patients had to be untreated at inclusion); positive thyroid peroxidase (TPO-Ab) and/or thyroglobulin (Tg-Ab) antibody concentrations according to local clinical guidelines; TSH concentrations >4.0 mIU/L; ability to complete paper-and-pencil questionnaires in Danish. Exclusion criteria were pregnancy and/or breastfeeding; thyroid cancer; previous radioiodine treatment or thyroid surgery; major comorbidities rendering completion of the study or interpretation of results improbable.

### Follow-up

All patients were treated and followed according to local clinical guidelines, with regular blood sampling and visits in the outpatient clinics. After initiation of LT4 therapy, the dosage was adjusted aiming for TSH levels within the reference ranges. At Odense University Hospital, a booklet containing two questionnaires (ThyPRO and SF-36 v2) and additional questions on sociodemographics, comorbidity, and non-thyroid medication was handed out at the first visit. At Rigshospitalet, the same booklet was sent to eligible patients; after two weeks a reminder was sent to non-responders. Patients completed the ThyPRO questionnaire prior to and six weeks and six months after initiation of treatment with LT4, and the SF-36 v2 questionnaire prior to and six months after commencement of therapy. ThyPRO data after six weeks of LT4 therapy was originally collected for methodological purposes in connection with the validation of the ThyPRO instrument [21], but provided extra data in this clinical study. For the same reason SF-36 was not collected in this cohort at six weeks. The questionnaires were collected by mail. Clinical data on diagnosis, treatment and biochemical variables were obtained by medical chart review. Biochemical variables included serum TSH (reference range: 0.3–4.0 mIU/L), total serum thyroxine (T4) (65–135 nmol/L), serum TPO-Ab (<30 mIU/L) and serum Tg-Ab (<20 mIU/L). Subclinical hypothyroidism was defined as TSH concentrations above the reference range with T4 within the reference range, and overt hypothyroidism as TSH above the reference range and T4 below the reference range.

A subset of the data has previously been used for a methodological evaluation of the responsiveness of the ThyPRO questionnaire [21].

### Outcomes

ThyPRO consists of 85 items on physical, mental and social domains of functioning and well-being in hypothyroidism, hyperthyroidism, non-toxic goiter, and Graves’ orbitopathy. The items employ a recall period of four weeks and are summarized in 13 multi-item scales and one single-item scale concerning Overall Impact of thyroid disease on HRQL. Each scale ranges 0–100, with higher scores indicating poorer health status. For specific items, please see the entire ThyPRO questionnaire (S1 Appendix).

The SF-36 consists of 36 items, also using a four week recall period, summarized in 8 scales. The scale scores can be further aggregated into physical and mental component summary scores. SF-36 scores were standardized using norm-based scoring to facilitate the comparison between SF-36 v1 (used in the general population sample) and v2 (used in the patient population). With norm-based scoring, mean and standard deviation (SD) is standardized to 50 and 10, respectively, in the general US population and higher scores indicate better health status.

### General population samples

ThyPRO data from the Danish general population were gathered from a random sample of adult citizens, using the Danish Civil Registration System, as previously described [22]. The questionnaire included items from the nine scales of the ThyPRO survey, which do not attribute HRQL-impact specifically to thyroid disease (in contrast to scales on impact of thyroid disease [Impaired Social Life, Daily Life, Sexlife, and Cosmetic Complaints]). The general population questionnaire also addressed socio-demographic variables, comorbidity, and medication. SF-36 data from the Danish general population were derived from the Danish Health Interview Survey in 2005, as described elsewhere [23,24]. For the present study we included general population data only for respondents living in the three regions from which patients were recruited.

### Statistical analysis

To address a potential selection bias, the available socio-demographic baseline characteristics of non-responders and responders were compared using the Wilcoxon-Mann-Whitney (age) and Chi-square (gender, education, chronic disease). Changes in mean HRQL scale scores and biochemical measurements between baseline and six weeks and six months after initiation of LT4 therapy were analyzed with the paired Student’s t-test. Multiple linear regression analyses, adjusted for age, gender, comorbidity and educational status were performed to test for differences in HRQL scale scores between patients and the general population samples, for associations between HRQL scale scores and TSH, T4 and TPO-Ab concentrations, respectively, and to compare baseline HRQL scale scores between participants completing follow-up surveys, and those lost to follow-up. Biochemical measurements within the four week recall period were used for analyses. The magnitude of differences between the general population and patient samples was evaluated by effect sizes, calculated as the mean difference divided by the pooled SD of the general population sample and the patients at baseline. The magnitude of changes in patient scores following treatment was evaluated by effect sizes calculated as the mean change divided by the SD in the patient sample at baseline. In accordance with Cohen, an effect size of 0.2–0.5 was defined as small, 0.5–0.8 as moderate and >0.8 as large [25]. *P*-values <0.05, were considered significant. All analyses were performed using SAS 9.4.

### Ethical considerations

According to Danish law, questionnaire studies do not require and thus cannot obtain approval by ethical committees. A completed, returned questionnaire is regarded as consent. The study was approved by the Danish Data Protection Agency (#2007-58-0015) and conducted in accordance with the Declarations of Helsinki.

## Results

### Participants

During 43 months, consecutive patients were screened and 100 patients with AIT were invited to participate. Seventy-eight patients completed the baseline (pre-treatment) survey (response rate 78%). In all, 63 patients completed follow-up surveys at six months, yielding a follow-up response rate of 81% of initially responding patients. The SF-36 was included in the surveys only after December 2009 and thus, 68 patients completed the SF-36 survey at baseline, and 58 (85% of initially responding patients) at six months. The median baseline age was 47 years, and 90% were females. All patients were treated with LT4 during the six-month study period. Socio-demographic characteristics, educational status, and comorbidity are shown in Table 1. There were no significant differences in baseline socio-demographic characteristics, biochemical measurements, or HRQL between the fifteen patients who completed only the baseline questionnaire and those completing the entire survey.

**Table 1:** Baseline sociodemographic characteristics of patients with autoimmune thyroiditis (AIT) and general population samples.

	AIT (n = 78)	General population ThyPRO (n = 739)	General population SF-36 (n = 6,638)
Women (n (%)):	70 (90)	602 (81)	3,605 (54)
Age in years (median (range))	47 (18–91)	50 (19–81)	50 (18–99)
Education (n (%))^a^			
≤10 years	10 (13)	129 (17)	1,133 (17)
11–14 years	25 (32)	313 (42)	3,679 (55)
≥15 years	34 (44)	249 (34)	1,695 (26)
No information	9 (11)	48 (7)	131 (2)
Chronic disease (n (%))^b^			
No chronic disease	53 (68)	499 (68)	4,024 (61)
1 chronic disease	18 (23)	165 (22)	1,706 (26)
≥2 chronic diseases	7 (9)	75 (10)	908 (14)

Table 1 legend:

^a^Combined school and professional education classified in accordance with the International Standard Classification of Education (www.uis.unesco.org).

^b^Includes: asthma, diabetes, ischaemic heart disease, stroke, chronic obstructive pulmonary disease, osteoarthritis, gastric/duodenal ulcer, anxiety and depression, other psychiatric diseases, chronic back pain and other conditions of the back.

### Biochemical measurements

At baseline, the median (interquartile range) TSH was 8.1 (5.5–12.2) mIU/L, T4 was 82 (72–99) nmol/L, and TPO-Ab was 526 (221–1590) mIU/L. One participant had negative TPO-Ab but positive Tg-Ab concentration at 68 mIU/L. At baseline, 66 patients had subclinical and 12 patients had overt hypothyroidism. After six weeks of LT4 therapy, serum TSH significantly decreased (*P*<0.0001) to a median (interquartile range) of 3.0 (1.7–4.4) mIU/L, further decreasing to 2.6 (1.4–4.2) mIU/L at six months. T4 significantly increased (*P*<0.0001), as compared with baseline, with median (interquartile range) concentrations of 104 (92–117) nmol/L and 102 (80–118) nmol/L after six weeks and six months, respectively. After six months, 24 patients still had elevated serum TSH, 21 of whom had subclinical hypothyroidism.

### HRQL

**Fig 1:**
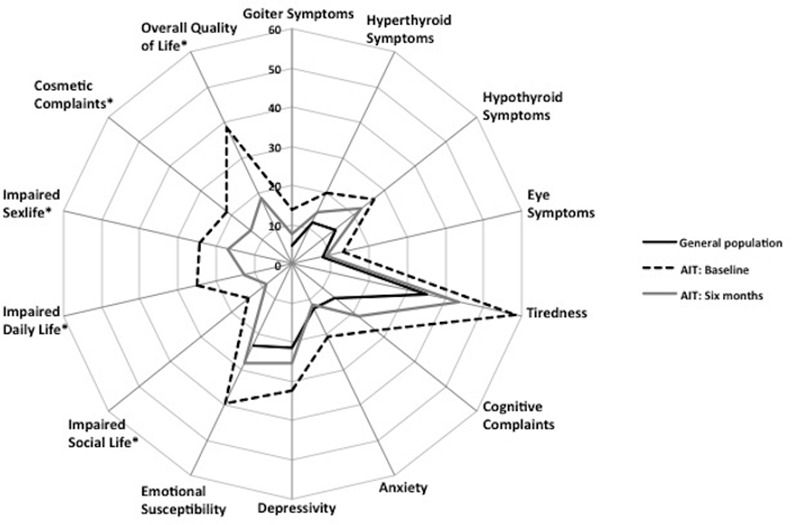
ThyPRO radar plot. Radar plot showing ThyPRO scale scores for patients with autoimmune hypothyroidism (AIT) at baseline and 6-months follow-up as well as scores from the general population sample. Each scale ranges 0–100, with higher scores indicating poorer quality of life. Items in ThyPRO scales marked* are asked with attribution to thyroid disease and cannot be answered by respondents from the general population.

**Fig 2:**
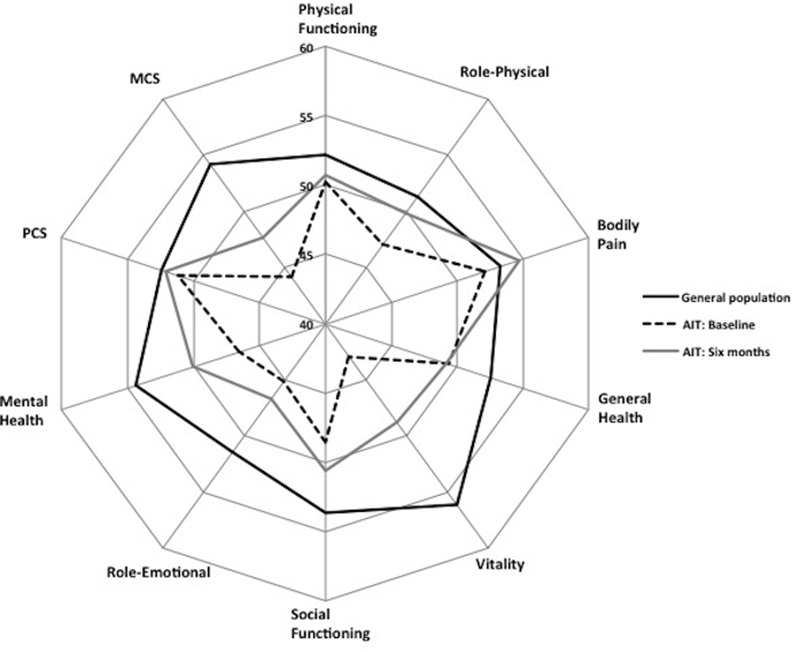
SF-36 Radar plot. Radar plot showing SF-36 scale scores for patients with autoimmune hypothyroidism (AIT) at baseline and 6-months follow-up as well as scores from the general reference population. Higher scores indicate better health status.

ThyPRO and SF-36 scale scores for patients at baseline and follow-up, and for general population samples are shown in Tables 2 and 3, and further illustrated in Figs 1 and 2, respectively.

**Table 2:** ThyPRO scale scores.

ThyPRO scales	General Population	Patients with autoimmune thyroiditis							
	(n = 739)	Baseline (n = 78)		Six weeks (n = 61)			Six months (n = 63)		
	Score	Score	Difference vs. GP	Score	Difference vs. GP	Change from baseline	Score	Difference vs. GP	Change from baseline
Goiter Symptoms	5 (9)	14 (15)	**9**+++	9 (12)	**4**+	**-4 (13)**+	8 (10)	**3**+	**-5 (13)**+
Hyperthyroid Symptoms	12 (14)	20 (16)	**8**++	14 (15)	2	**-5 (13)**+	14 (13)	2+	**-4 (12)**+
Hypothyroid Symptoms	14 (16)	27 (24)	**13**++	25 (23)	**11**++	-1 (18)	23 (21)	**9**++	-2 (16)
Eye Symptoms	8 (11)	13 (14)	**5**++	9 (10)	1	**-3 (9)**+	9 (11)	1	**-3 (9)**+
Tiredness	35 (21)	58 (28)	**23**+++	47 (26)	**12**++	**-10 (23)**+	43 (27)	**8**+	**-12 (23)**+
Cognitive Complaints	14 (17)	27 (26)	**13**++	22 (23)	**8**+	-3 (23)	22 (23)	**8**+	-3 (19)
Anxiety	13 (16)	21 (20)	**8**+	13 (14)	0	**-6 (17)**+	11 (17)	-2	**-7 (15)**+
Depressivity	21 (18)	32 (23)	**11**++	27 (20)	6+	-4 (18)	25 (19)	4+	**-5 (20)**+
Emotional Susceptibility	23 (19)	40 (25)	**17**+++	29 (24)	6+	**-8 (20)**+	28 (22)	5+	**-10 (18)**+
Impaired Social Life	-	13 (19)	-	10 (15)	-	**-4 (16)**+	8 (15)	-	**-5 (17)**+
Impaired Daily Life	-	22 (27)	-	14 (21)	-	**-8 (18)**+	12 (20)	-	**-9 (20)**+
Impaired Sexlife	-	23 (30)	-	16 (25)	-	**-6 (22)**+	17 (23)	-	-6 (31)+
Cosmetic Complaints	-	20 (21)	-	17 (23)	-	-4 (17)	14 (18)	-	**-6 (16)**+
Overall HRQL	-	37 (36)	-	25 (28)	-	**-13 (30)**+	19 (26)	-	**-18 (34)**++

**Table 2 legend and notes:** Mean (SD) ThyPRO scale scores (0–100, higher scores indicating worse HRQL) of the general population (GP) sample and patients with autoimmune thyroiditis at baseline (before treatment) and at follow-up (six weeks and six months after initiation of LT4 therapy). Differences between patients and the general population sample were analyzed with multiple linear regression analysis, adjusting for age, sex, comorbidity, and educational status. Changes between baseline and follow-up for patients completing the questionnaire at both assessments were analyzed with the paired t-test. Discrepancies between mean scores and mean changes were due to the 15 responders lost to follow-up. Magnitudes of changes were evaluated by effect sizes (mean difference/SD_baseline_). Items in five ThyPRO scales (Impaired Social Life, Impaired Daily Life, Impaired Sexlife, Cosmetic Complaints, and Overall Quality of Life) are asked with attribution to thyroid disease and cannot be answered by respondents from the general population. Statistically significant differences (P<0.05) are marked in bold.

+Small effect size (0.2–0.5)

++Moderate effect size (0.5–0.8)

+++Large effect size (>0.8), according to Cohen et al. [25].

**Table 3:** SF-36 scale scores.

SF-36 scales	GeneralPopulation	Patients with autoimmune thyroiditis				
	(n = 6,638)	Baseline (n = 68)		Six months (n = 58)		
	Score	Score	Difference vs GP	Score	Difference vs GP	Change from baseline
Physical Functioning	52 (8)	50 (8)	**-2**+	51 (9)	-1	0 (6)
Role-Physical	51 (9)	47 (10)	**-4**+	50 (9)	-1	**3 (8)**+
Bodily Pain	53 (10)	52 (12)	-1	55 (10)	2	3 (9)+
General Health	53 (9)	49 (10)	**-4**+	49 (11)	-4+	1 (6)
Vitality	56 (10)	43 (13)	**-13**+++	49 (13)	**-7**++	**6 (11)**+
Social Functioning	54 (8)	49 (12)	**-5**++	51 (11)	**-3**+	3 (8)+
Role-Emotional	51 (9)	45 (12)	**-6**++	47 (12)	**-5**++	2 (11)
Mental Health	54 (9)	47 (9)	**-7**+++	50 (10)	**-4**++	**4 (8)**+
**SF-36 summary scores**						
Physical Component	51 (9)	51 (10)	0	52 (8)	1	1 (6)
Mental Component	54 (8)	44 (12)	**-10**+++	48 (11)	**-6**++	**4 (9)**+

**Table 3 legend and notes:** Mean (SD) norm-based SF-36 scale scores (higher scores indicating better HRQL) of the general population sample (GP) and patients with autoimmune thyroiditis at baseline (before treatment) and at follow-up (six months after initiation of LT4 therapy). Differences between patients and the GP sample were analyzed with multiple linear regression analysis, adjusting for age, sex, comorbidity, and educational status. Changes between baseline and follow-up for patients completing the questionnaire at both assessments were analyzed with the paired t-test. Discrepancies between mean scores and mean changes were due to the 10 responders lost to follow-up. Magnitudes of changes were evaluated by effect sizes (mean difference/SD_baseline_). Statistically significant differences (P<0.05) are marked in bold.

+Small effect size (0.2–0.5)

++Moderate effect size (0.5–0.8)

+++Large effect size (>0.8), according to Cohen et al. [25].

#### Comparison with general population samples before treatment

Baseline ThyPRO scores among patients were significantly higher (worse) than the general population scores in all nine comparable scales (*p*-values <0.0001). Differences varied, from small effect sizes for Eye Symptoms and Anxiety to large effect sizes for Goiter Symptoms, Tiredness, and Emotional Susceptibility. The most severely impaired scale was the Tiredness scale (effect size: 1.05). Patients’ baseline SF-36 scores were lower (worse) than those in the general population sample in seven of eight scales, with effect sizes ranging from small to large, except for the Bodily Pain scale, where there was no difference. Large differences were seen in the Mental Health and Vitality scales, and the Mental Component summary score, with the largest difference for Vitality (effect size 1.37). There was no difference between patients and the general population sample in the SF-36 Physical Component summary.

#### Changes following medical therapy

Scores significantly decreased (improved) for nine of thirteen ThyPRO scales as well as for overall HRQL after six weeks of therapy. All improvements, except Impaired Sexlife, were consistent after six months of therapy with additional significant improvements observed for the Depressivity and Cosmetic Complaints scales. All significant changes in the ThyPRO multi-item scales were of small magnitude, while the overall HRQL item improved with a moderate effect size, after six months of therapy (p<0.0001).

Six months after initiation of LT4 therapy, the SF-36 scales for Role-Physical, Bodily Pain, Vitality, Social Functioning and Mental Health, and the Mental Component summary score, had improved significantly, all with small effect sizes.

#### Comparison with general population samples at follow-up

According to ThyPRO, there were no longer any large differences after six weeks of treatment, as compared with the general population samples. However, significant moderate differences persisted in physical Hypothyroid Symptoms and Tiredness, as did small differences in Goiter Symptoms and Cognitive Complaints. After six months, the same four scales remained significantly different from the general population sample. Only the Hypothyroid Symptoms scale attained a moderate effect size at this time point, while the other deficits were of small magnitude.

After six months of LT4 therapy several of the SF-36 scales, i.e. Mental Health, Role-Emotional, Social Functioning, Vitality, and the Mental Component, were still significantly impaired compared to the general population, showing moderate to small effect sizes.

### Associations between biochemical measurements and HRQL

There were no significant associations between serum levels of TSH, total T4 or TPO-Ab and any of the ThyPRO or SF-36 scores, neither at baseline nor during follow-up. Neither were there any differences in the scores or effect of treatment, when comparing patients with subclinical (n = 66) versus overt hypothyroidism (n = 12) at baseline or follow-up.

## Discussion

In this prospective cohort study, we have assessed HRQL in patients with AIT. For the first time HRQL has been measured longitudinally in hypothyroid patients with both validated disease-specific (ThyPRO) and generic (SF-36) surveys, and compared with HRQL data from general population samples. Prior to LT4 therapy, we found significant impacts of varying magnitude across disease-specific (ThyPRO) and generic (SF-36) HRQL aspects, as compared to general population samples. Impairments ranged from large differences for Tiredness to small differences for Physical Functioning, while Bodily Pain was the only unaffected scale. The wide array of impacts was corroborated in a recent population-based case-control study of newly diagnosed overt AIT, with tiredness being the most frequently reported symptom [26]. Since volunteer control subjects also experienced many symptoms indicative of hypothyroidism, the authors found that neither presence nor absence of individual symptoms is reliable for diagnosis of the disease [26]. In addition, there were no associations between symptom burden and biochemical markers at disease onset [26]. Thus, tiredness may be reported irrespective of disease or biochemical disease severity. Nevertheless, our study quantifies that tiredness is severely impacted in newly diagnosed AIT patients, as compared with the general population. After six weeks of treatment, we found a partial remission of the disease-specific tiredness, which further ameliorated at six months. However, a small to moderate impairment persisted, also when measured by the SF-36 Vitality scale. Thus, rather than reporting tiredness as a present/absent symptom, as is the classical diagnostic medical approach, quantifying it on a continuum, as done with ThyPRO, offers a more detailed picture of the course of disease symptomatology.

Deficits persisted for a subset of other disease-specific and generic scales. The ThyPRO scale showing the largest deficit at six months was the physical Hypothyroid Symptoms scale, including items such as “Have you been sensitive to cold?” and “Have you had dry skin?” This scale, along with Cognitive Complaints, did not respond to treatment during the study period. However, it has been suggested that persisting cognitive complaints, even in patients with subclinical hypothyroidism (the majority of our participants), are most likely an independent entity requiring separate evaluation [27]. Another aspect of particular interest is the poorly understood relationship between hypothyroidism and depression [28]. In a recent study both depressive symptoms and sexual dysfunction were related to subclinical hypothyroidism in female patients with AIT [29]. We found depressivity moderately impacted in the untreated state, but this item improved during follow-up and was not significantly different, as compared with the general population, at the end of the study. The severity of sexual dysfunction cannot be compared with the general population in our study, as items in the ThyPRO scale for sexual impairments are asked with attribution to thyroid disease, and therefore irrelevant to the general population.

Many disease-specific issues significantly improved already within six weeks, with further improvements after six months, when the majority of SF-36 scales were also improved. All significant improvements were of small magnitude, except the ThyPRO overall HRQL item that improved with a moderate effect size at six months. This item asks the question: “During the past four weeks, has your thyroid disease had a negative effect on your quality of life?” It could be speculated that small improvements in various aspects accumulate, and lead to a larger overall effect.

Interestingly, we found similar impact and treatment effects when comparing patients with subclinical and overt hypothyroidism, supporting that also patients with mild biochemical thyroid failure benefit from treatment, at least from a HRQL point of view. This study was implemented in the daily-life clinic, reflected by the fact that approximately 30% of the patients had a TSH level above the reference range after six months of therapy. This is well in line with the Colorado thyroid disease prevalence study, where 40% of patients taking thyroid medication had abnormal TSH levels [30]. Thus, suboptimal LT4 dose or poor patient compliance seems to be the rule and not unique to our study. It could be speculated that obtaining euthyroidism might have resulted in more pronounced HRQL improvements. However, we found no associations between thyroid function and HRQL in our regression analyses, and previous observations also suggest that normalization of TSH with LT4 does not safeguard against persistent complaints [3]. Here, biochemical data, used for the regression analyses, were obtained by medical chart review. Future long-term studies should prospectively standardize the timing between measurement of HRQL and serum markers of thyroid function, in order to further elaborate the relationship between biochemical dysfunction and HRQL.

Strengths of our study are its longitudinal design, the use of validated disease-specific and generic questionnaires, and inclusion of general population reference groups. This conceptual framework can readily be applied to the study of quality of life in other diseases. However, there are also limitations. Although individuals lost to follow-up had similar sociodemographic characteristics they tended to have more impaired HRQL, albeit not statistically significant, as compared with patients who had a full survey. Three of fifteen patients (20%) lost to follow-up had overt hypothyroidism at baseline, while this number was 9/63 (14%) in patients with a full survey (9/63). This, as well as recruitment solely from university hospital outpatient clinics, may have induced selection bias. Generally, there was consistency between significance tests and effect sizes, but a minority of small differences or changes did not reach statistical significance, which may be caused by lack of power. We considered applying formal corrections for multiple comparisons, but decided not to, because the results were consistent [31] with multiple comparisons pointing towards the same conclusion (illustrated by Figs 1 and 2), and to avoid type II errors. Lack of power may explain the absence of associations between the degree of thyroid dysfunction and HRQL, as e.g. only 12 patients had overt hypothyroidism at baseline. Finally, although hypothyroidism–also when adequately treated–is associated with increased morbidity and mortality [32–34], we cannot rule out that HRQL issues would have improved further with longer follow-up. Extended follow-up could also have helped to unmask a potential response bias of participants knowingly being started on LT4. Disfavoring such a bias, the improvements at six weeks were, with one exception, consistent at six months.

Despite the limitations mentioned, our data can help clinicians inform hypothyroid patients what to expect, when starting on LT4 therapy. Benefits of addressing the patient perspective in medical communication are well established [35] and future studies should investigate whether HRQL assessment in clinical practice can improve the care for hypothyroid patients [36]. Recent investigations encourage initiatives that stimulate ethical placebo mechanisms in clinical practice. HRQL assessment could do so by contributing, in a positive manner, to the overall therapeutic context [37]. To advance its applicability, both in clinical practice and research, we have recently developed a short version of ThyPRO [38]. It constitutes the primary outcome in our on-going randomized controlled trial of selenium supplementation in AIT [39], and can be put to ideal use in future trials investigating various formulations of LT4 and/or combinations with liothyronine [3] or placebo-controlled trials in subclinical hypothyroidism [28].

In conclusion, untreated hypothyroid patients experience widely impacted HRQL, compared to the general population. Most aspects improve during the first six months of LT4 therapy but some deficits remain, including tiredness, which is the cardinal manifestation. Whether full remission in HRQL, with this or any combination of LT4 and liothyronine, will ensue with longer follow-up remains to be explored.

## Supporting Information

S1 Appendix ThyPROus questionnaire. (PDF)
