# Supplementary material for: Disease-Specific as Well as Generic Quality of Life Is Widely Impacted in Autoimmune Hypothyroidism and Improves during the First Six Months of Levothyroxine Therapy
Source: PLoS One. 2016 Jun 3;11(6):e0156925. doi: 10.1371/journal.pone.0156925 (PMC4892657; doi:10.1371/journal.pone.0156925)
Supplement: S1 Appendix — (PDF) [file pone.0156925.s001.pdf]

Quality of Life Questionnaire for Patients with Thyroid Disease

-ThyPROus-

This questionnaire is about how your thyroid disease has affected your life.

Please answer each question by marking ☒ by the answer that best fits you. If you are unsure about how you want to answer, please give the best answer you can.

Copyright © Torquil Watt, Copenhagen University Hospital Rigshospitalet, Denmark

The first section of the questionnaire is about symptoms, tiredness, memory, mood, and health.

Please base your **answers on how you have been feeling <u>in general</u>** during the past 4 weeks.

1. ***The first questions are about symptoms***

	**During the past 4 weeks have you**	Not at all	A little	Some	Quite a bit	Very much
1a	- had the sensation of fullness in the neck?	☐	☐	☐	☐	☐
1b	- had a <u>visible</u> swelling in the front of your neck?	☐	☐	☐	☐	☐
1c	- felt pressure in your throat?	☐	☐	☐	☐	☐
1d	- had pain in the front of your throat?	☐	☐	☐	☐	☐
1e	- had pain in your neck that could be felt in your ears?	☐	☐	☐	☐	☐
1f	- had the sensation of a lump in your throat?	☐	☐	☐	☐	☐
1g	- had the need to clear your throat frequently?	☐	☐	☐	☐	☐
1h	- felt discomfort swallowing?	☐	☐	☐	☐	☐
1i	- had difficulty swallowing?	☐	☐	☐	☐	☐
1j	- had the sensation of suffocating?	☐	☐	☐	☐	☐
1k	- been hoarse?	☐	☐	☐	☐	☐
1l	- had trembling hands?	☐	☐	☐	☐	☐
1m	- had a tendency to sweat a lot?	☐	☐	☐	☐	☐
1n	- experienced palpitations (rapid heart beat)?	☐	☐	☐	☐	☐
1o	- experienced shortness of breath?	☐	☐	☐	☐	☐
1p	- been sensitive to heat?	☐	☐	☐	☐	☐
1q	- been sensitive to cold?	☐	☐	☐	☐	☐
1r	- had an increased appetite?	☐	☐	☐	☐	☐

Copyright © Torquil Watt, Copenhagen University Hospital Rigshospitalet, Denmark

	During the past 4 weeks have you	Not at all	A little	Some	Quite a bit	Very much
1s	- had loose stools?	☐	☐	☐	☐	☐
1t	- had an upset stomach?	☐	☐	☐	☐	☐
1u	- had moist or watery eyes?	☐	☐	☐	☐	☐
1v	- had bags under the eyes or swollen eyelids?	☐	☐	☐	☐	☐
1w	- had the sensation of dryness or “grittiness” in the eyes?	☐	☐	☐	☐	☐
1x	- had impaired vision?	☐	☐	☐	☐	☐
1y	- felt pressure in (or behind) the eyes?	☐	☐	☐	☐	☐
1z	- had double vision?	☐	☐	☐	☐	☐
1aa	- had eye pain?	☐	☐	☐	☐	☐
1bb	- been very sensitive to light?	☐	☐	☐	☐	☐
1cc	- had swollen hands or feet?	☐	☐	☐	☐	☐
1dd	- had dry skin?	☐	☐	☐	☐	☐
1ee	- had itchy skin?	☐	☐	☐	☐	☐

2. ***The following questions are about tiredness***

	During the past 4 weeks have you	Not at all	A little	Some	Quite a bit	Very much
2a	- been tired?	☐	☐	☐	☐	☐
2b	- been exhausted?	☐	☐	☐	☐	☐
2c	- had difficulty getting motivated to do anything at all?	☐	☐	☐	☐	☐
2d	- felt worn out?	☐	☐	☐	☐	☐

Copyright © Torquil Watt, Copenhagen University Hospital Rigshospitalet, Denmark

3. ***The following questions are about your vitality***

**During the past 4 weeks have you**		Not at all	A little	Some	Quite a bit	Very much
3a	- felt full of life?	☐	☐	☐	☐	☐
3b	- felt energetic?	☐	☐	☐	☐	☐
3c	- been able to cope with the demands of your life?	☐	☐	☐	☐	☐

4. ***The following questions are about memory and concentration***

**During the past 4 weeks have you**		Not at all	A little	Some	Quite a bit	Very much
4a	- had difficulty remembering?	☐	☐	☐	☐	☐
4b	- had slow or unclear thinking?	☐	☐	☐	☐	☐
4c	- had difficulty finding the right words?	☐	☐	☐	☐	☐
4d	- been confused?	☐	☐	☐	☐	☐
4e	- had difficulty learning something new?	☐	☐	☐	☐	☐
4f	- had difficulty concentrating?	☐	☐	☐	☐	☐

Copyright © Torquil Watt, Copenhagen University Hospital Rigshospitalet, Denmark

5. *The following questions are about nervousness and tension*

		**During the past 4 weeks have you**	Not at all	A little	Some	Quite a bit	Very much
5a		- felt nervous?	☐	☐	☐	☐	☐
5b		- felt afraid or anxious?	☐	☐	☐	☐	☐
5c		- felt tense?	☐	☐	☐	☐	☐
5d		- been concerned about being seriously ill?	☐	☐	☐	☐	☐
5e		- felt uneasy?	☐	☐	☐	☐	☐
5f		- felt restless?	☐	☐	☐	☐	☐

6. *The following questions are about psychological well-being*

	**During the past 4 weeks have you**	Not at all	A little	Some	Quite a bit	Very much
6a	- felt sad?	☐	☐	☐	☐	☐
6b	- felt depressed?	☐	☐	☐	☐	☐
6c	- felt discouraged?	☐	☐	☐	☐	☐
6d	- cried easily?	☐	☐	☐	☐	☐
6e	- felt unhappy?	☐	☐	☐	☐	☐

	**During the past 4 weeks have you**	Not at all	A little	Some	Quite a bit	Very much
6f	- felt happy?	☐	☐	☐	☐	☐
6g	- had self-confidence?	☐	☐	☐	☐	☐

Copyright © Torquil Watt, Copenhagen University Hospital Rigshospitalet, Denmark

7. ***The following questions are about having difficulty coping or having mood swings***

**During the past 4 weeks have you**	Not at all	A little	Some	Quite a bit	Very much
7a - had difficulty coping?	☐	☐	☐	☐	☐
7b - felt “not like yourself”?	☐	☐	☐	☐	☐
7c - noticed you easily felt stressed?	☐	☐	☐	☐	☐
7d - had mood swings?	☐	☐	☐	☐	☐
7e - felt irritable?	☐	☐	☐	☐	☐
7f - felt frustrated?	☐	☐	☐	☐	☐
7g - felt angry?	☐	☐	☐	☐	☐

	Not at all	A little	Some	Quite a bit	Com-pletely
7h - felt in control of your life?	☐	☐	☐	☐	☐
7i - felt in balance?	☐	☐	☐	☐	☐

Copyright © Torquil Watt, Copenhagen University Hospital Rigshospitalet, Denmark

The remainder of the questionnaire is about **how your thyroid disease may have affected various aspects of your life**

8. ***The following questions are about your relationships with other people***

		**During the past 4 weeks, <u>has your thyroid disease caused</u> you to**	Not at all	A little	Some	Quite a bit	Very much
8a	- have difficulty being together with other people (for example, spouse, children, boy/girlfriend, friends, or others)?	☐	☐	☐	☐	☐
8b	- feel you were a burden to other people?	☐	☐	☐	☐	☐
8c	- have conflicts with other people?	☐	☐	☐	☐	☐

	**During the past 4 weeks have you**	Not at all	A little	Some	Quite a bit	Very much
8d	- felt that people in your surroundings have lacked understanding of your thyroid disease?	☐	☐	☐	☐	☐

Copyright © Torquil Watt, Copenhagen University Hospital Rigshospitalet, Denmark

9. ***The following questions are about your daily activities***

	**During the past 4 weeks, <u>has your thyroid disease caused</u> you to**	Not at all	A little	Some	Quite a bit	Very much
9a	- have difficulty managing your daily life?	☐	☐	☐	☐	☐
9b	- limit your leisure activities or hobbies?	☐	☐	☐	☐	☐
9c	- not be able to participate in life around you?	☐	☐	☐	☐	☐
9d	- have difficulty getting around (for example, walking, running, bicycling, or driving a car)?	☐	☐	☐	☐	☐
9e	- feel as if everything takes longer to do?	☐	☐	☐	☐	☐

	**During the past 4 weeks, <u>has your thyroid disease caused</u> you to**	I do not work	Not at all	A little	Some	Quite a bit	Very much
9f	- have <u>difficulty managing your job</u> (for example, finding it hard to cope or calling in sick)?	☐	☐	☐	☐	☐	☐

Copyright © Torquil Watt, Copenhagen University Hospital Rigshospitalet, Denmark

10. ***The following questions are about your sex life***

During the past 4 weeks have you	Not at all	A little	Some	Quite a bit	Very much
10a - felt your thyroid disease had a negative influence on your sex life?	☐	☐	☐	☐	☐
10b - had a decreased sexual desire?	☐	☐	☐	☐	☐

11. ***Thyroid diseases (or their treatment) may affect your <u>appearance</u>. (For example, by causing swelling of the neck, swollen face, hands, or feet, or changes in weight or to the eyes.)***

During the past 4 weeks,	Not at all	A little	Some	Quite a bit	Very much
11a - has your thyroid disease <u>affected your appearance</u> (for example, swelling of the neck, eye changes, weight changes)?	☐	☐	☐	☐	☐
11b - have you been <u>unsatisfied</u> with your appearance because of your thyroid disease?	☐	☐	☐	☐	☐
11c - have you tried to <u>camouflage or mask</u> visible signs of your thyroid disease (for example, by wearing a scarf or sunglasses)?	☐	☐	☐	☐	☐
11d - have you been bothered by other people looking at you?	☐	☐	☐	☐	☐
11e - has your thyroid disease influenced which clothes you wear?	☐	☐	☐	☐	☐
11f - has your thyroid disease made you feel too fat?	☐	☐	☐	☐	☐

Copyright © Torquil Watt, Copenhagen University Hospital Rigshospitalet, Denmark

12. ***The final question is about to what extent your thyroid disease has affected you overall during the past 4 weeks***

**During the past 4 weeks,**	Not at all	A little	Some	Quite a bit	Very much
12 - has your thyroid disease had a negative effect on your quality of life?	☐	☐	☐	☐	☐

Please go back and check that you have answered all the questions.

Thank you very much for your help answering this questionnaire!

Copyright © Torquil Watt, Copenhagen University Hospital Rigshospitalet, Denmark
